# Decoding the Genomic Variability among Members of the *Bifidobacterium* *dentium* Species

**DOI:** 10.3390/microorganisms8111720

**Published:** 2020-11-03

**Authors:** Gabriele Andrea Lugli, Chiara Tarracchini, Giulia Alessandri, Christian Milani, Leonardo Mancabelli, Francesca Turroni, Vera Neuzil-Bunesova, Lorena Ruiz, Abelardo Margolles, Marco Ventura

**Affiliations:** 1 Laboratory of Probiogenomics, Department of Chemistry, Life Sciences, and Environmental Sustainability, University of Parma, 43124 Parma, Italy; chiara.tarracchini@studenti.unipr.it (C.T.); christian.milani@unipr.it (C.M.); leonardo.mancabelli@unipr.it (L.M.); francesca.turroni@unipr.it (F.T.); 2 Department of Veterinary Medical Science, University of Parma, 43126 Parma, Italy; giulia.alessandri1@studenti.unipr.it; 3 Microbiome Research Hub, University of Parma, 13121 Parma, Italy; 4 Department of Microbiology, Nutrition and Dietetics, Czech University of Life Sciences Prague, Kamycka 129, 16500 Prague, Czech Republic; bunesova@af.czu.cz; 5 Department of Microbiology and Biochemistry, Dairy Research Institute of Asturias, Spanish National Research Council (IPLA-CSIC), Paseo Río Linares s/n, Villaviciosa, 33300 Asturias, Spain; lorena.ruiz@ipla.csic.es (L.R.); amargolles@ipla.csic.es (A.M.); 6 MicroHealth Group, Instituto de Investigación Sanitaria del Principado de Asturias (ISPA), Oviedo, 33011 Asturias, Spain

**Keywords:** bifidobacteria, genomics, pangenome, phylogeny

## Abstract

Members of the *Bifidobacterium dentium* species are usually identified in the oral cavity of humans and associated with the development of plaque and dental caries. Nevertheless, they have also been detected from fecal samples, highlighting a widespread distribution among mammals. To explore the genetic variability of this species, we isolated and sequenced the genomes of 18 different *B. dentium* strains collected from fecal samples of several primate species and an *Ursus arctos*. Thus, we investigated the genomic variability and metabolic abilities of the new *B. dentium* isolates together with 20 public genome sequences. Comparative genomic analyses provided insights into the vast metabolic repertoire of the species, highlighting 19 glycosyl hydrolases families shared between each analyzed strain. Phylogenetic analysis of the *B. dentium* taxon, involving 1140 conserved genes, revealed a very close phylogenetic relatedness among members of this species. Furthermore, low genomic variability between strains was also confirmed by an average nucleotide identity analysis showing values higher than 98.2%. Investigating the genetic features of each strain, few putative functional mobile elements were identified. Besides, a consistent occurrence of defense mechanisms such as CRISPR–Cas and restriction–modification systems may be responsible for the high genome synteny identified among members of this taxon.

## 1. Introduction

For millennia, animals and bacteria have coevolved and coadapted to establish interspecies relationships [1]. In this context, the gut microbiota composition of humans has been studied to identify which species grew together with their host and their role in human health [2,3]. Among hundreds of bacterial species that inhabit humans, members of the *Bifidobacterium* genus were identified as one of the dominant colonizers of the human gut [2]. These microorganisms were classified as Gram-positive, nonspore-forming, and nonmotile bacteria that belong to the phylum Actinobacteria [4]. To date, 88 different bifidobacterial (sub)species were classified, mostly isolated from the gastrointestinal tract of numerous animals, the human gut and oral cavity, and insect hindgut [5,6,7,8,9,10,11,12,13,14,15,16,17,18,19].

Among members of this genus, the *Bifidobacterium dentium* species was usually identified in the oral cavity of humans and associated with the development of plaque and dental caries, and might transiently pass the human intestine [20,21,22,23,24,25]. Recently, a study aiming to explore the distribution of bifidobacteria in fecal samples of a wide range of animals highlighted their widespread distribution across the mammalian kingdom [26]. In this framework, *B. dentium* was not shown to be a predominant bifidobacterial species, while *Bifidobacterium adolescentis*, *Bifidobacterium bifidum*, *Bifidobacterium longum,* and *Bifidobacterium pseudolongum* were found widely distributed among mammals. Nonetheless, its occurrence was identified in various mammalian orders, such as Carnivora, Artiodactyla, Perissodactyla, and Primates [26]. While comparative genomic analyses of most abundant bifidobacterial taxa identified in the human gut have been performed [27,28,29,30,31] for *B. dentium* species, little information exists concerning its genomic characterization.

Another intriguing characteristic of this bifidobacterial species is the ability to produce γ-aminobutyric acid (GABA), which is a neuroactive molecule involved in the modulation of neural signals that affect neurological and psychiatric parameters as well as sleep, appetite, mood, and cognition [32,33]. This peculiar physiological signature has been found in each *B. dentium* genome sequenced to date [33], while the sensory neuron activity in a rat fecal retention model of visceral hypersensitivity has been proved by oral administration of *B. dentium* GABA-producers [34]. Furthermore, it has been recently shown that *B. dentium* can enhance the production of the host’s mucin by upregulation of gene expression and autophagy signaling pathways [35].

Among publicly available *B. dentium* strains, only three genomes were isolated from nonhuman samples but from species of *Bradypus*. Thus, we decided to explore the genome variability of this taxon by sequencing 18 newly isolated *B. dentium* strains from fecal samples belonging to several species of primates and an *Ursus arctos*. Resulting genomic sequences were employed to achieve a comprehensive comparative genome analysis involving 38 different strains. A phylogenetic investigation was undertaken using the core genome sequences of this taxon, allowing to unveil a high level of genomic conservation among the analyzed strains. Furthermore, the genomic complexity of the taxon was further investigated to outline genetic features involved in the preservation of the chromosomal integrity, such as clustered regularly interspaced short palindromic repeats (CRISPR) and restriction–modification (RM) systems.

## 2. Materials and Methods

### 2.1. Bifidobacterial Genome Sequences

Three complete genome sequences belonging to *B. dentium* strains were retrieved from the National Center for Biotechnology Information (NCBI) public database. In addition, partial genome sequences of 17 *B. dentium* strains were also retrieved from NCBI to investigate the genetic variability of the species. Furthermore, the genome sequence of 18 newly isolated *B. dentium* strains were decoded and deposited at DDBJ/ENA/GenBank under accession numbers listed in Table 1 (BioProject PRJNA666310), together representing a collection of 38 *B. dentium* genomes.

### 2.2. Isolation of B. dentium Strains

One gram of each fecal sample was mixed with 9 mL of phosphate-buffered saline (PBS), pH 6.5. Serial dilutions and successive plating were performed using de Man–Rogosa–Sharpe (MRS) agar (Scharlau Chemie, Barcelona, Spain), supplemented with 0.05% (*w*/*v*) L-cysteine hydrochloride and 50 μg/mL mupirocin (Delchimica, Italy). The addition of the latter chemicals was used to enhance the selective growth of members of the *Bifidobacterium* genus. Agar plates were incubated in a chamber (Concept 400; Ruskin, Bristol, UK) with an anaerobic atmosphere (2.99% H_2_, 17.01% CO_2_, and 80% N_2_) at 37 °C for 48 h. Among fecal samples reported in this study, about 1000 colonies were selected and subcultivated in MRS broth supplemented with 0.05% (*w*/*v*) L-cysteine hydrochloride in an anaerobic chamber at 37 °C for 16 h. Morphologically different colonies grown on MRS plates were randomly picked and restreaked to isolate purified bacterial strains. Then, DNA was extracted from all isolates and characterized as previously described [36]. Specifically, DNA was submitted to a *B. dentium* species-specific identification using a PCR methodology with primers Bdent1 5′-ATCCCGGGGGTTCGCCT-3′ and Bdent2 5′-GAAGGGCTTGCTCCCGA-3′, which had been designed on the 16S rRNA gene sequence of this taxon. PCR amplification was performed according to the following protocol: one cycle of 94 °C for 5 min, followed by 30 cycles of 94 °C for 30 s, 54 °C for 30 s and 72 °C for 50 s, and finally one cycle of 72 °C for 5 min. This procedure allowed the identification of 32 strains belonging to the *B. dentium* species estimating a charge of *B. dentium* on the selective media of 3.2%.

### 2.3. Microbial DNA Extraction

*B. dentium* strains were inoculated in de Man–Rogosa–Sharpe (MRS) (Scharlau Chemie, Barcelona, Spain) medium to perform chromosomal DNA extraction. The latter medium was supplemented with 0.05% (*w*/*v*) L-cysteine hydrochloride and incubated at 37  °C in an anaerobic atmosphere (2.99% (*v*/*v*) H_2_, 17.01% (*v*/*v*) CO_2_, and 80% (*v*/*v*) N_2_) employing an anaerobic chamber (Concept 400, Ruskin). Cells from 10 mL of an overnight culture were harvested by centrifugation at 6000 rpm for 8 min. The obtained cell pellet was used for DNA extraction using the GenElute^TM^ Bacterial Genomic DNA kit (Sigma-Aldrich, Darmstadt, Germany) following the manufacturer’s guidelines. Then, internal transcribed spacer (ITS) sequences were amplified from extracted DNA using primer pair Probio-bif_Uni/Probio-bif_Rev [37] and sequenced to avoid the decoding of clonal strains. Finally, sequenced ITS sequences were compared to a public database composed of bifidobacterial ITS sequences (http://probiogenomics.unipr.it/pbi/) involving the basic local alignment search tool (BLAST). This procedure allowed for discarding clonal strains within the same sample and to reduce the number of different *B. dentium* strains from 32 to 18. Table 1 lists the isolated strains employed in this study.

### 2.4. B. dentium Chromosomal Sequencing and Assemblies

The chromosomal DNA of 18 *B. dentium* strains was decoded by GenProbio Srl (http://genprobio.com) using a MiSeq platform (Illumina, San Diego, CA, USA) according to the supplier’s protocol as previously described [30] by using the Nextera XT DNA Library Prep Kit (Illumina). The library samples obtained were then pooled into a Flow Cell V3 600 cycle (Illumina). Fastq files of paired-end reads generated from each genome sequencing effort were used as input for the genome assembly through the MEGAnnotator pipeline [38]. The SPAdes program v3.14.0 was used for de novo assembly of each bifidobacterial genome sequence with the pipeline option “--careful” and a list of k-mer sizes 21, 33, 55, 77, 99, 127 [39]. MEGAnnotator then employed contigs greater than 1000 bp to predict protein-encoding open reading frames (ORFs) using Prodigal [40]. Predicted ORFs were then functionally annotated using RAPSearch2 (reduced alphabet based protein similarity search) (cutoff e-value of 1 × 10^−5^ and minimum alignment length 20) employing the NCBI reference sequences (RefSeq) database [41] together with hidden Markov model profile (HMM) searches (http://hmmer.org/) performed against the manually curated Pfam-A database (cutoff e-value of 1 × 10^−10^). Then, tRNA genes were detected through tRNAscan-SE v1.4 [42], while rRNA genes were identified by means of RNAmmer v1.2 [43]. To guarantee a consistent genomic analysis, ORFs from each *B. dentium* genome sequence retrieved from NCBI public databases were repredicted and reannotated employing the identical bioinformatics pipeline used for the 18 *B. dentium* strains isolated in this study. The presence of extrachromosomal DNA was investigated using a curated database of plasmid sequences downloaded from NCBI [44] and using the tool mlplasmids v1.0 [45].

### 2.5. Comparative Genomic Analysis

The genome content of the 38 reconstructed *B. dentium* strains (Table 1) was submitted to a pangenome calculation by means of the pan-genomes analysis pipeline (PGAP) [46]. Predicted proteome of a specific *B. dentium* strain was screened for orthologues against the proteome of the other collected *B. dentium* strain employing BLAST analysis [47] (cutoff e-value of <1 × 10^−5^ and 50% identity over at least 80% of both protein sequences). The data obtained were then clustered into protein families, also named as clusters of orthologous groups (COGs), employing MCL (graph theory-based Markov clustering algorithm) [48] by means of the method gene family (GF). Then, using the PGAP software, a pangenome profile was built based on a presence/absence matrix including all identified COGs of the 38 analyzed strains. The core genome of the *B. dentium* species was then defined based on protein families shared among each analyzed genome. Furthermore, truly unique genes (TUGs) encoded by a single analyzed genome that are not present in other *B. dentium* chromosomes were also identified.

### 2.6. Phylogenetic and Phylogenomic Analyses

The concatenated sequence of amino acids belonging to the core genome of each *B. dentium* was aligned by means of the MAFFT software [49], and the resulting phylogenetic trees was built using the neighbor-joining method through the ClustalW v2.1 program [50]. Then, the graphical viewer of phylogenetic trees FigTree v1.4 (http://tree.bio.ed.ac.uk/software/figtree/) was used to build a *B. dentium* phylogenetic tree. A value of average nucleotide identity (ANI) was calculated using the program fastANI using each genome pair as input [51].

### 2.7. Genomic Analyses

The proteome of each *B. dentium* strain was screened for genes predicted to encode carbohydrate-active enzymes based on sequence similarity to genes classified in the carbohydrate-active enzyme (CAZy) database [52]. Each predicted proteome of a given bifidobacterial strain was screened for orthologues against data of 17,538 bacterial genomes using HMMER v3.3 [53] (cutoff e-value of 1 × 10^−15^) and BLASTp analysis [47] (cutoff e-value of 1 × 10^−30^). Thus, a screening was performed employing the dbCAN2 meta server [54], followed by a BLASTp validation of the obtained results. The values of the glycosyl hydrolases (GH) index were attributed to dividing the number of GH counts of each bifidobacterial type strain against the total number of its predicted genes (Table 1). The presence of genes encoding restriction enzymes was performed using the REBASE database [55]. The prediction of clustered regularly interspaced short palindromic repeats (CRISPR) and associated Cas-encoding genes was achieved through CRISPRCasFinder v4.2.2 [56]. The presence of genes that are predicted to be acquired by horizontal gene transfer (HGT) events was performed using COLOMBO v4.0 [57], with a sensitivity value of 0.4 as previously applied to type strains of the genus *Bifidobacterium* [58]. Identification of prophagelike elements was assessed using a custom database composed of bifidobacterial genes previously classified as prophage genes from each type strain [59]. Then, predicted prophages were further profiled by means of the PHAge Search Tool Enhanced Release (PHASTER) [60]. Each predicted proteome was also screened for the presence of insertion-sequence (IS) elements through the IS Finder online tool (https://isfinder.biotoul.fr/). Predicted genes were manually evaluated to remove false positives from each genomic analysis. *B. dentium* proteome was screened for proteins that can act as antibiotic resistance proteins by employing the MEGARes database through a BLASTp analysis (cutoff e-value of 1 × 10^−30^) [61]. Putative bacteriocin-encoding genes were predicted using BAGEL3 with default parameters [62]. Toxin–antitoxin systems were predicted using the TADB v2.0 database through a BLASTp analysis (cutoff e-value of 1 × 10^−5^) [63].

### 2.8. Carbohydrate Growth Assays

*B. dentium* strains were cultivated on semisynthetic MRS medium supplemented with a 0.5% (*w*/*v*) concentration of a specific sugar, and the optical densities (measured at a wavelength of 600 nm) were recorded using a plate reader (BioTek, Winooski, VT, USA). The plate was read in intermittent mode, with absorbance readings performed at 3 min intervals for three times after 48 h of growth, where each reading was ahead of 30 s of shaking at medium speed. Cultures were grown in biologically independent triplicates, and the reported growth data were expressed as the means of these replicates. Carbohydrates used in this study were acquired from Sigma and Carbosynth (Berkshire, UK).

### 2.9. Statistical Analyses

Hierarchical clustering (HCL) analyses were performed by using TM4 MeV software [64], and cladograms were visualized through FigTree software (http://tree.bio.ed.ac.uk/software/figtree/).

## 3. Results and Discussion

### 3.1. Genomic Overview of the B. dentium Taxon

Many published studies discussed the peculiar ecological niche of *B. dentium*, highlighting the occurrence of this taxon with dental caries [20,21,22,23,24]. However, a genomic comparison between the sequenced *B. dentium* strains is somewhat limited, and comparative genome analyses characterizing the genomic diversity and genetic features of members of this species have not been described so far. Thus, in the framework of a previous ecologic survey of bifidobacterial communities in mammals, using a culture-dependent approach, we isolated 17 different *B. dentium* strains from fecal samples of primates and one single strain from a fecal sample of *Ursus arctos* (Table 1) [26]. More specifically, we isolated *B. dentium* strains from seven primate species, i.e., *Colobus guereza*, *Eulemur macaco*, *Lemur catta*, *Macaca silenus*, *Pan troglodytes*, and *Pongo borneo*, as well as *Homo sapiens* (Table 1).

Accordingly, a next-generation sequencing (NGS) approach was used to decode the genomes of the 18 newly isolated *B. dentium* strains. The sequences obtained were then analyzed together with 20 additional publicly available *B. dentium* genomes listed in Table 1. Genomic sequences of newly isolated *B. dentium* strains were sequenced to a coverage depth that ranged from 40-fold to 255-fold, which upon assembly resulted in 10 to 37 contigs (Table 1). By using the complete genome of the type strain *B. dentium* DSM 20436 as the reference sequence (NCBI accession number AP012326.1), we predicted the contig orientation for each draft genome. After the assembly, the genome length of each *B. dentium* strain was retrieved, resulting in genomes whose size was shown to range from 2,431,695 to 2,697,950 bp (Table 1). Overall, the number of predicted open reading frames (ORFs) ranged from 1936 for *B. dentium* VB29 to 2371 for *B. dentium* BIOML-A2 (Table 1). Thus, collected general genome features may suggest a noticeable genome variability at first glance, with a variation of more than 200 Kbps and 400 ORFs between genomes. Notably, the genome variability identified between strains was not correlated with the presence of extrachromosomal DNA, since no plasmid sequences were identified among *B. dentium* chromosomes.

### 3.2. Pangenome and Core Genome of the B. dentium Species

**Figure 1:**
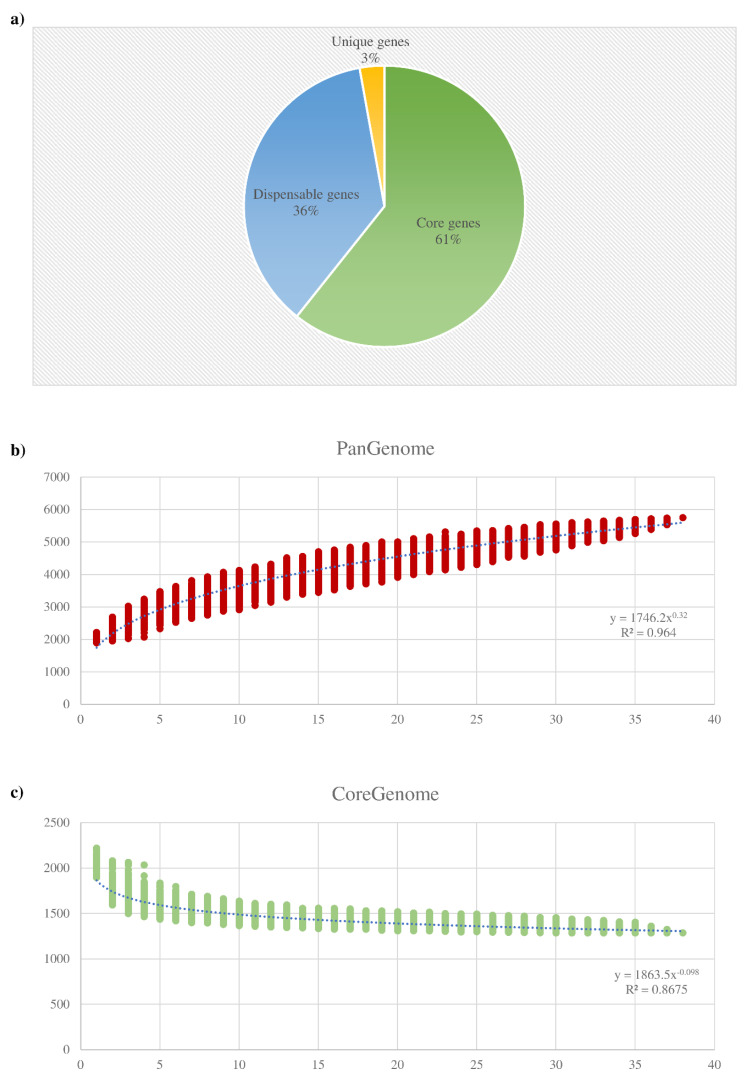
*B. dentium* pangenome and core genome: (**a**) displays the number of core genes (green), unique genes (yellow), and dispensable genes (blue) identified in the pangenome analysis; (**b**) shows the pangenome size based on the sequential addition of the 38 *B. dentium* genomes; (**c**) illustrates the core genome size based on the sequential addition of the same genomes.

Comparative genome analysis of the *B. dentium* species was performed employing identified genes of each collected strain. Accordingly, the pangenome analysis of the *B. dentium* taxon was undertaken using a previously described method based on the generation of clusters of orthologous groups (COGs) [65]. The latter analysis allowed the identification of 5754 COGs, representing the pangenome of the *B. dentium* species. Accordingly, 1287 COGs of the *B. dentium* pangenome were shared among the 38 genomes of this taxon, thus representing the so-called core genome of the *B. dentium* species corresponding to 22.4% of its pangenome (Figure 1).

Moreover, genes shared between two or more strains, i.e., dispensable genes, and genes present in just one of the analyzed strains, i.e., truly unique genes (TUGs), were also identified. This analysis allowed us to detect a number of TUGs that ranged from 19 for *B. dentium* BIOML-A3 to 219 for *B. dentium* 793B, with an average of 60 TUGs per genome (Table 1). Thus, the average TUG number highlighted that the variability of this taxon was sufficiently explored since the *B. dentium* ORFeome contains a small number of TUGs when compared to the previously analyzed bifidobacterial pangenomes of *B. adolescentis* and *B. bifidum* [27,28].

The pangenome size, when plotted versus the 38 *B. dentium* genomes, shows that the power trendline tends to reach a plateau. As reported in Figure 1, the genomic data from the last strain added to the pangenome analysis slightly expand the total gene pool. Thus, in accordance to the reported data, the resulting pangenome curve suggests a “closed” *B. dentium* pangenome. More specifically, after the addition of the 38th *B. dentium* genome, any additional genome included in the analysis will result in the increases of the pangenome size of about 60 COGs (Figure 1).

The extended core genome coupled with the relatively small number of TUGs and the prediction of a “closed” pangenome, suggested that this taxon does not possess a high genome variability in respect to other bifidobacterial species previously scrutinized, such as *Bifidobacterium asteroides*, *B. longum,* and *B. pseudolongum* [6,30,66]. Instead, the variability between *B. dentium* strains resulted in being comparable with that previously inspected in members of the *B. bifidum* and *Bifidobacterium breve* species [27,67].

### 3.3. Phylogenetic Analyses and Evolutionary Development of the B. dentium Taxon

**Figure 2:**
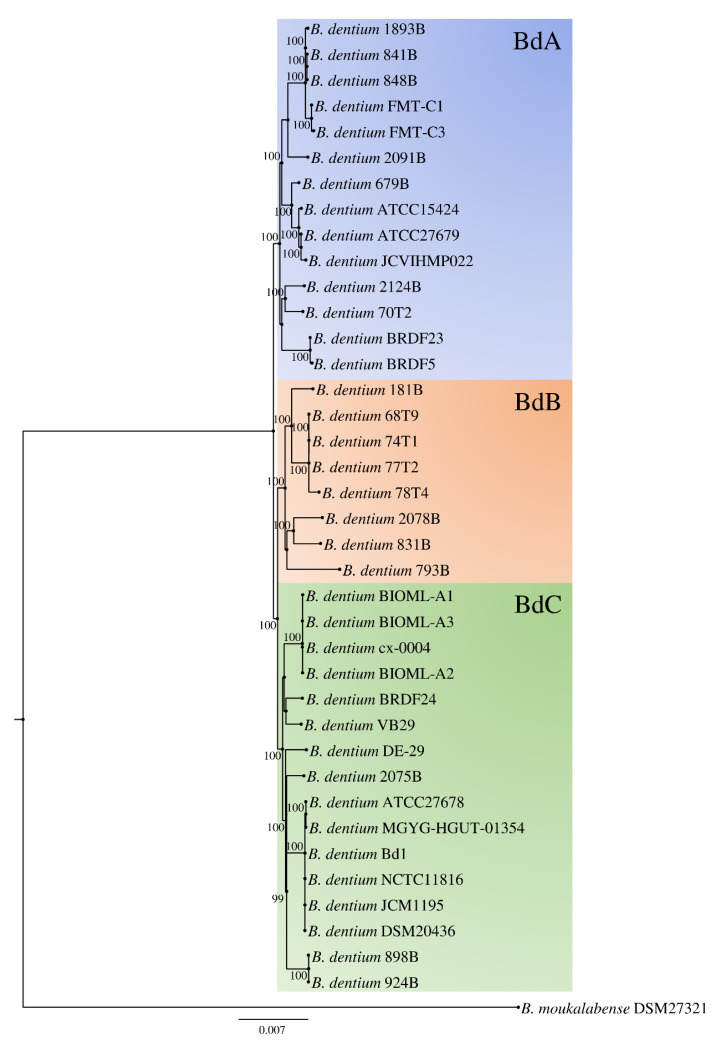
Phylogenetic tree of the *B. dentium* species. The proteomic tree is based on a concatenation of 1140 core genes identified in the pangenome analysis of 38 *B. dentium* strains. Phylogenetic groups are highlighted in different colors. The tree was constructed by the neighbor-joining method, and the genome sequence of *Bifidobacterium moukalabense* DSM 27321 was used as an outgroup. Bootstrap percentages above 50 are shown at node points, based on 1000 replicates.

The assessment of the *B. dentium* phylogeny was performed applying a previously described methodology [58,68,69] by using data collected from the comparative genomic analysis reported above. Thus, gene sequences of the 38 *B. dentium* strains were employed as well as those of *Bifidobacterium moukalabense* DSM 27321, which served as an outgroup. Then, paralogs were excluded from the 1287 *B. dentium* core genes identified in the core genome analysis using the PGAP pipeline (see Materials and Methods), resulting in 1140 genes of which the concatenated amino acid sequences were employed to build a phylogenetic tree (Figure 2). The resulting *B. dentium*-based phylogenetic tree showed the presence of three clusters named BdA, BdB, and BdC, composed by 14, 8, and 16 strains, respectively, which seem to have a very close phylogenetic relatedness (Figure 2).

*B. dentium* strains that were isolated from human beings did not fit into a specific cluster, as well as strains isolated from the other species of primates. Conversely, *B. dentium* strains isolated from different mammals were found evenly distributed among all clusters. Thus, the reconstructed *B. dentium*-based phylogenetic tree highlighted that none of the 38 strains considered in this study presented divergent genetic signatures among its core genome sequence. Unfortunately, few genome sequences belonging to strains isolated from the oral cavity were publicly available. So, all four *B. dentium* retrieved from this ecological niche included in the phylogenetic tree represented the type strain of this species (Figure 2).

To further explore the overall genomic variability among *B. dentium*, we employed the average nucleotide identity (ANI) approach between all collected genome sequence pairs. Accordingly, we highlighted the genome synteny among members of this taxon, with associated ANI values ranging from 98.2% to 99.9% (Supplementary Materials Table S1). Notably, two strains that display an ANI value <95% may be considered to belong to two distinct species [70]. Based on this notion, in our previous *Bifidobacterium* based phylogenomic studies, we identified a suitable ANI threshold of about 94% to discriminate between bifidobacterial species [6,68,69]. Thus, ANI values above 98.2% obtained from this phylogenomic analysis highlighted a low variability between genomes of members of the *B. dentium* taxon.

### 3.4. Carbohydrate-Active Enzymes among Members of the B. dentium Species

**Figure 3:**
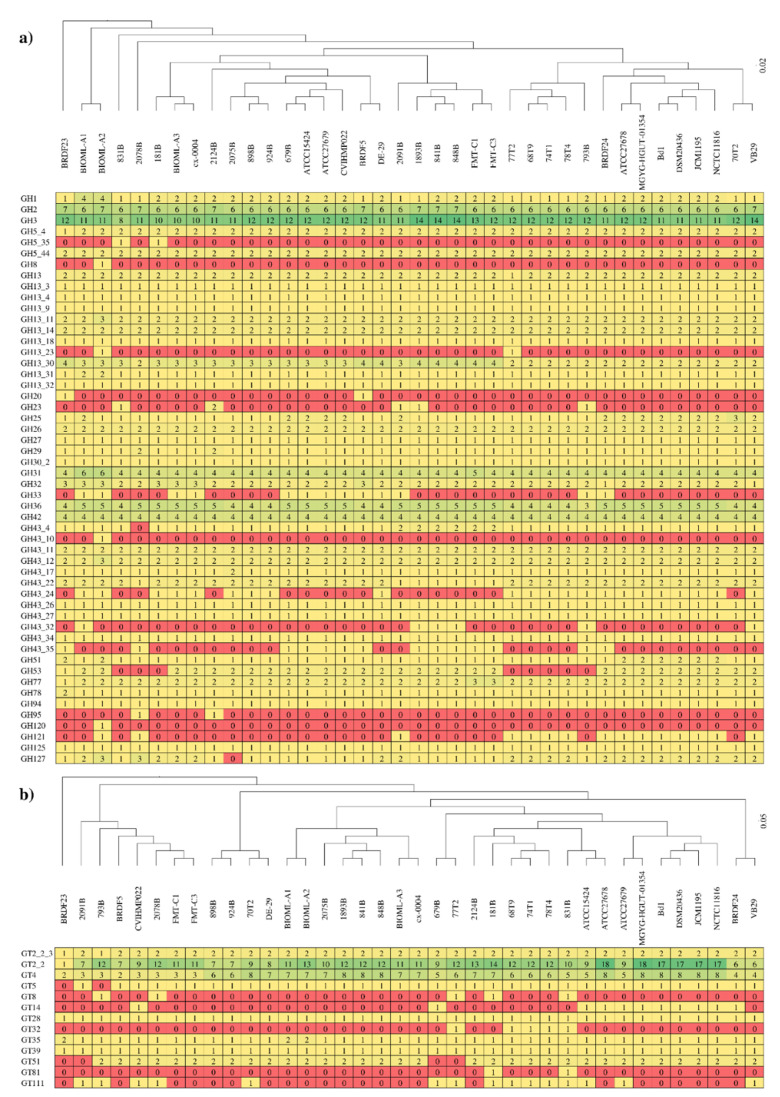
Carbohydrate-active enzymes of the *B. dentium* taxon: (**a**) displays a heat map of the glycosyl hydrolases (GHs) prediction among 38 *B. dentium* strains analyzed, while (**b**) exhibits a heat map of the predicted glycosyltransferases (GTs) of the same genomes. Colors reflect the abundance of the identified genes, starting from zero (red) to 18 (green). On top of both panels, hierarchical clustering is reported based on the identified GH and GT families retrieved in each strain.

The complete carbohydrate-active enzyme repertoire of the *B. dentium* taxon was explored to unveil predicted genes encoding glycosyl hydrolases (GHs) and glycosyltransferases (GTs) of each analyzed strain. The screening allowed us to identify members of 29 GH families and 12 GT families, highlighting a predominance of genes predicted to encode GHs belonging to the GH13, GH43, GH3, and GH2 families, and GTs of the GT2 and GT4 families (Figure 3). Together with GH5, GH36, GH31, GH26, and other minority families, which were also identified in the glycobiome of each *B. dentium*, these GH families represent the wide core glycobiome of the *B. dentium* taxon involving 19 different GH families. Notably, the most abundant GH family identified in *B. dentium*, represented by GH13, is also widespread in bacteria and it is characterized in the degradation of a wide range of carbohydrates, including plant-derived polysaccharides, such as amylose, amylopectin, maltodextrins, and pullulan. The degradative abilities of this taxon related to plant-derived polysaccharides were also confirmed by the other most abundant GHs, i.e., GH43 and GH3, involved in the degradation of complex plant polysaccharides, such as (arabino)xylan, (arabino)galactan, and other arabinans. Furthermore, the high number of GH2 family enzymes were represented by β-galactosidases and β-mannosidase, highlighting an advanced ability into the degradation of simple carbohydrates as well. Nonetheless, genes encoding additional 15 GH families are shared among each analyzed *B. dentium* strain, highlighting a massive degradative ability towards a wide range of carbohydrates (Figure 3). Additionally, each analyzed genome revealed a conserved distribution of genes encoding for carbohydrate esterases (CEs), while no polysaccharide lyases (PLs) were identified. More specifically, members of four CE families were identified in each *B. dentium* genome, i.e., CE1, CE2, CE4, and CE9, showing a distribution of one to three family genes per genome.

To identify differences between GH profiles of each *B. dentium* strain, hierarchical clustering was performed using the complete repertoire of GHs and GTs identified. As disclosed in Figure 3, the resulting clusters did not show any appreciable differences in the GH and GT family profiles. Furthermore, no correspondence was found between phylogenetic clustering (BdA, BdB, and BdC clusters of Figure 2) and clustering based on GHs or GTs families (Figure 3). Remarkably, *B. dentium* BRDF23 strain was reported as the outlier of both carbohydrate-active enzymes clustering, but any peculiar gene encoding carbohydrate-active enzymes arise from the analysis.

In order to compare the amount of genetic material involved in the carbohydrate metabolism, the GH count of each *B. dentium* strain was normalized with the number of predicted genes. The latter process generated GH indexes ranging from 3.59% in *B. dentium* 831B to 4.24% in *B. dentium* VB29, highlighting a conserved GH distribution among strains. As previously observed, *B. dentium* DSM 20436 possesses the highest GH index of the genus *Bifidobacterium* together with other species isolated from the gut of different primates [68,71]. Thus, the vast repertoire of identified GHs, corroborate with the ability of members of this taxon to establish a relationship with the host in multiple compartments, such as the oral cavity rich in simple sugars and the gut, where the host has not processed complex carbohydrates.

The carbohydrate fermentation capabilities of the *B. dentium* taxon were then investigated through growth experiments involving newly *B. dentium* isolated strains together with the type strain of this species. To obtain a complete as possible overview into the *B. dentium* metabolic abilities, we included both simple and complex plant-derived glycans as the unique carbon source on a semisynthetic medium. As displayed in Figure S1, each *B. dentium* strain grew on common simple sugars, such as glucose and galactose. Furthermore, all strains could metabolize a broader array of complex sugars, such as maltodextrin, starch, and xylan. However, analyzed strains displayed little if any growth on arabinogalactan, arabinose, and N-acetyl-D-glucosamine (Figure S1). As previously observed through the in silico carbohydrate-active enzyme profiling, each *B. dentium* strains’ growth performance resulted in a conserved pattern between glycans, except for growths on mannose (Figure S1).

### 3.5. B. dentium Mobilome

To investigate the amount of putative horizontal gene transfer (HGT) events that occurred among the *B. dentium* taxon, the genome of the 38 strains were screened using the software Colombo [57,58]. The analysis resulted in identifying an average of 8.4% putative genes involved in HGT events with respect to the total amount of *B. dentium* predicted genes (Table 1). Thus, members of this species were found to be less suitable to acquire alien genes with respect to the same analysis recently applied to members of the *B. pseudolongum* subsp. *globosum* taxon [30]. This characteristic was also reflected by the low number of TUGs previously identified (Table 1).

As previously shown through comparative genomic analysis of numerous species belonging to the *Bifidobacterium* genus, prophage sequences and other elements involved in the transposition of genes, such as transposases, represent a large part of the genetic material implicated in HGT events [58]. Thus, we performed a prophage profiling taking into account the pangenome of the *B. dentium* taxon, by using a manually curated database composed by genes of prophages identified among the *Bifidobacterium* genus [59]. Thus, 75 prophage sequences were identified, ranging from zero to three between *B. dentium* chromosomes (Table 1). In this context, retrieved prophage sequences were predicted to belong to the *Siphoviridae* family, encompassing modules that putatively encode functions involved in lysogeny, DNA replication, DNA packaging, head and tail morphogenesis, and host lysis [72]. Nonetheless, an additional classification of the latter sequences using the PHASTER tool, classified them as incomplete prophages. Nevertheless, each reported phage sequences displayed several representative genes of the modules listed above, such as integrases, capsid, portal and terminase proteins, tape measure proteins, and lysin and holing proteins (Table S2).

A further investigation into the *B. dentium* mobilome involved a screening of insertion sequence (IS) elements employing the IS Finder online tool. Overall, IS21 and IS3 were those IS families retrieved from the proteome of each strain, highlighting a few occurrences of one or two IS elements per genome, while in half of these genomes, no IS elements were identified (Table 1). Nevertheless, the *B. dentium* DE-29 strain was the only exception, displaying four IS elements in its genome (Table 1). Furthermore, across the analyzed genomes, many additional genes were predicted to encode transposases, but we observed a high frequency of partial IS elements that are no longer functional, showing an evolutionary development of this taxon aimed to avoid genome rearrangements.

### 3.6. B. dentium Defense Mechanisms

To enhance their fitness, (bifido)bacteria evolved to contrast HGT events through the ability to defend themselves against the invasion of foreign DNA [73]. To explore the defense mechanism of each *B. dentium* strain, we investigated the presence of CRISPR–Cas systems, as previously reported for each type-strains of the genus *Bifidobacterium* [74]. Among the 38 genomes analyzed, one or two CRISPR–Cas systems were identified in each *B. dentium* strain, except for the 2091B strain. Altogether, 46 CRISPR–Cas systems were shared among the 38 *B. dentium* chromosomes, highlighting a high occurrence of type I systems (I-C and I-U) characterized by the *cas3* gene, and type II systems (II-C) by the *cas9* gene (Table 1 and Table S3). Based on our screening, type III systems characterized by the *cas10* gene appear to be absent in our assessed strain collection. Interestingly, type II systems were found in 11 *B. dentium* strains in possession by another type I system. Thus, among the *B. dentium* taxon, we observed a high degree of conservation of type I systems employed by this taxon to defend themselves from phage invasions.

Another defense mechanism complex that prevents the acquisition of foreign DNA is represented by restriction–modification (RM) systems [75]. The screening revealed that the type IV RM system represented the predominant RM system among the *B. dentium* taxon, being present in 31 out of 38 genomes, followed by the type II RM system identified in the genomes of 22 strains (Table 1 and Table S4). Furthermore, type I and III RM systems were identified at a lower frequency, i.e., in 16 and 4 strains, respectively (Table S4). Additional RM genes were retrieved from this analysis but were not identified in specific gene clusters and were excluded from the classification (Table S4). Thus, members of the *B. dentium* species encoded an average of two putative functional RM systems aiming to prevent the invasion by foreign DNA sequences. In this context, only *B. dentium* 70T2 lacked in RM systems (Table 1). Altogether, these findings revealed conserved defense mechanisms in almost the entire *B. dentium* collection, proving a direct correlation between a reduced mobilome and an efficient defense against the invasion of foreign DNA. Correlation between phylogenetic grouping, as reported in Figure 2, and mobilome/defense mechanisms profiles of each strain were not supported by any statistical significance. Thus, note that members of the *B. dentium* taxon share a close phylogenetic relatedness with low variability between genomes.

Predicted protein-coding genes were then investigated for the presence of antibiotic resistance (AR) elements. However, putative AR genes encoding transporters were omitted from this analysis due to the inaccuracy of their bioinformatic prediction [76,77]. Thus, false positives were excluded through manual editing of the results based on the protein domains identified within predicted AR genes. The overall number of AR genes was of two putative AR enzymes in each of the 38 *B. dentium* genomes acting against beta-lactams and tetracyclines. More specifically, each *B. dentium* possesses in its core genome a transglycosylase characterized by a penicillin-binding domain, and a translation elongation factor with a TetM-like domain. In the same fashion, predicted protein-coding genes were screened for the presence of bacteriocins able to inhibit the growth of other bacteria. However, the analyzed proteome of the *B. dentium* taxon lacked in these peptidic toxins. Furthermore, the presence of toxin–antitoxin systems was also investigated. The screening highlighted 177 putative toxin–antitoxin (TA) protein-encoding genes shared among the 38 *B. dentium* chromosomes (Table S5). The large majority of the putative TA genes were identified to be widespread across the *B. dentium* genomes, while 60 were closely linked, representing actual type II TA systems. The most conserved system was represented by the HipAB system, identified in 25 out of 38 *B. dentium* strains (Table S5). Additional TA systems were represented by three RelBE systems identified in *B. dentium* BIOML-A1 and *B. dentium* BIOML-A2, and two HicAB systems identified in *B. dentium 793B* and *B. dentium 2078B* (Table S5).

## 4. Conclusions

Since their discovery, members of the *B. dentium* species have been associated with the development of plaque and dental caries in humans [20,21,22,23]. In the last few years, however, a wider distribution of this taxon in the fecal samples of several mammals has been shown [26]. Based on this notion, through culture dependent isolation attempts, we reconstructed the genome sequences of 17 *B. dentium* strains identified in the gut microbiota of primates and one single strain from a fecal sample of *Ursus arctos*. Comparative genomic analyses and phylogenetic reconstruction of the taxon revealed that it does not possess a high genome variability regarding other bifidobacterial species, highlighting a very close phylogenetic relatedness between strains. In order to better understand this notion, we explored the mobilome and the defense mechanisms shared between members of the *B. dentium* species, resulting in identification of a few transposable elements within their genome coupled with consistent machinery to defend against the invasion of foreign DNA. Furthermore, the profiling of the carbohydrate-active enzyme repertoire of each analyzed strain highlighted a taxon that is evolved with a consistent wide core glycobiome aimed to degrade an equally wide range of carbohydrates.

## Figures and Tables

**Table 1:** General genome features of *B. dentium* strains.

Strain	Host	Isolation Source	Cluster	Number of Bases	COVERAGE	Contigs	GC Content	ORFs	rRNA loci	tRNA	TUGs	GHs Number	GH Index (%)	HGT (%)	Transposase	Prophages	RM Systems	CRISPR System	Accession Number
*Newly sequenced genomes*																			
68T9	*Macaca silenus*	Stool sample	BdB	2467165	74	13	58.43	1997	4	55	23	77	3.9	7	0	2	1	TypeIU	PRJNA666310
70T2	*Pan troglodytes*	Stool sample	BdA	2487785	96	35	58.31	2046	4	57	114	78	3.8	7.6	2	3	0	TypeIU	PRJNA666310
74T1	*Eulemur macaco*	Stool sample	BdB	2469902	40	15	58.43	2005	4	55	26	77	3.8	7.3	0	2	1	TypeIU	PRJNA666310
77T2	*Lemur catta*	Stool sample	BdB	2472493	70	37	58.43	2012	4	56	33	77	3.8	7.3	0	2	1	TypeIU	PRJNA666310
78T4	*Colobus guereza*	Stool sample	BdB	2468941	44	17	58.41	2007	4	55	31	77	3.8	7.4	0	2	1	TypeIU	PRJNA666310
181B	*Homo sapiens*	Stool sample	BdB	2523858	99	10	58.45	2066	4	56	65	78	3.8	9.7	0	1	3	TypeIC	PRJNA666310
679B	*Homo sapiens*	Stool sample	BdA	2697950	42	15	58.46	2255	3	56	85	81	3.6	9.3	2	3	3	-	PRJNA666310
793B	*Homo sapiens*	Colonoscopy	BdB	2605010	73	19	58.7	2178	4	53	219	79	3.6	9.6	1	2	2	TypeIC	PRJNA666310
831B	*Homo sapiens*	Colonoscopy	BdB	2479317	40	14	58.38	2007	4	56	77	72	3.6	7.4	0	2	2	TypeIU	PRJNA666310
841B	*Homo sapiens*	Stool sample	BdA	2580247	53	25	58.38	2090	4	56	22	84	4.0	7.6	1	1	2	TypeIIC/TypeIU	PRJNA666310
848B	*Homo sapiens*	Colonoscopy	BdA	2582570	122	21	58.35	2091	4	56	25	84	4.0	7.8	1	1	2	TypeIIC/TypeIU	PRJNA666310
898B	*Homo sapiens*	Colonoscopy	BdC	2459079	40	24	58.49	1971	4	57	27	78	4.0	5.6	0	0	3	TypeIU	PRJNA666310
924B	*Homo sapiens*	Colonoscopy	BdC	2456361	97	15	58.5	1966	4	55	23	78	4.0	5.4	0	0	3	TypeIU	PRJNA666310
2075B	*Pongo pygmaeus*	Stool sample	BdC	2531372	255	14	58.64	2078	3	57	100	77	3.7	9.9	0	2	1	TypeIIC/TypeIIA	PRJNA666310
2078B	*Pongo pygmaeus*	Stool sample	BdB	2612253	211	19	58.57	2140	4	58	74	79	3.7	11.3	2	1	1	TypeIE	PRJNA666310
2091B	*Pongo pygmaeus*	Stool sample	BdA	2537119	218	34	58.68	2084	4	56	86	81	3.9	10.9	1	3	3	-	PRJNA666310
2124B	*Ursus arctos*	Stool sample	BdA	2627726	120	14	58.59	2120	4	55	68	82	3.9	8.4	2	0	2	TypeIC	PRJNA666310
VB29	*Macaca silenus*	Stool sample	BdC	2431695	76	14	58.46	1936	4	55	74	82	4.2	7.5	1	1	3	TypeIC	PRJNA666310
*Public genomes*																			
1893B	*Homo sapiens*	Stool sample	BdA	2571068	-	24	58.22	2070	4	56	47	84	4.1	8.4	2	0	3	TypeIIC/TypeIU	NAQE.1
ATCC15424	*Homo sapiens*	Pleural fluid	BdA	2624481	-	19	58.48	2174	4	56	77	81	3.7	8.4	1	2	3	TypeIU	SQQK.1
ATCC27678	*Homo sapiens*	Stool sample	BdC	2642081	-	2	58.52	2137	4	56	24	82	3.8	8	0	2	2	TypeIC/TypeIIC	ABIX.2
ATCC27679	*Homo sapiens*	Vaginal tract	BdA	2644565	-	8	58.45	2200	n.p.	58	34	81	3.7	9.5	1	2	2	TypeIU	AEEQ.1
Bd1	*Homo sapiens*	Dental caries	BdC	2636367	-	1	58.54	2142	4	56	28	81	3.8	8.2	0	2	2	TypeIC/TypeIIC	CP001750.1
BIOML-A1	*Homo sapiens*	Stool sample	BdC	2752113	-	88	58.22	2288	3	60	98	88	3.8	10	1	1	3	TypeIC	WDPC.1
BIOML-A2	*Homo sapiens*	Stool sample	BdC	2868671	-	100	58.09	2371	4	61	130	96	4.0	9.9	1	1	3	TypeIC	WDPD.1
BIOML-A3	*Homo sapiens*	Stool sample	BdC	2590214	-	13	58.46	2130	4	53	19	80	3.8	10.5	0	1	2	TypeIC	WDPE.1
BRDF5	*Bradypus*	Stool sample	BdA	2629155	-	16	58.59	2147	n.p.	53	26	82	3.8	9.4	2	0	1	TypeIU	VYSF.1
BRDF23	*Bradypus*	Stool sample	BdA	2557123	-	14	58.5	2068	4	54	20	81	3.9	8.3	1	0	1	TypeIU	VYSE.1
BRDF24	*Bradypus*	Stool sample	BdC	2511344	-	22	58.64	2049	3	55	82	80	3.9	8.1	0	1	1	TypeIC	VYSD.1
JCVIHMP022	*Homo sapiens*	Urogenital tract	BdA	2633876	-	31	58.45	2257	n.p.	56	142	81	3.6	9.1	1	2	2	TypeIU	AEHJ.1
cx-0004	*Homo sapiens*	Stool sample	BdC	2589878	-	19	58.46	2129	4	53	20	80	3.8	10.3	0	1	2	TypeIC	RCXJ.1
DE-29	*Homo sapiens*	Stool sample	BdC	2551991	-	25	58.53	2088	4	55	107	80	3.8	8.2	4	0	2	TypeIC	BCYE.1
DSM20436	*Homo sapiens*	Dental caries	BdC	2668067	-	2	58.55	2177	4	56	42	81	3.7	7.7	0	2	2	TypeIC/TypeIIC	FNSE.1
FMT-C1	*Homo sapiens*	Stool sample	BdA	2538385	-	29	58.49	2146	4	56	70	83	3.9	7.6	1	1	2	TypeIIC/TypeIU	JAAWWM0.1
FMT-C3	*Homo sapiens*	Stool sample	BdA	2538722	-	32	58.49	2156	4	54	79	81	3.8	7.5	1	1	2	TypeIIC/TypeIU	JAAWWN0.1
JCM1195	*Homo sapiens*	Dental caries	BdC	2635669	-	1	58.54	2141	4	56	24	81	3.8	8.2	0	2	2	TypeIC	AP012326.1
MGYG-HGUT-01354	*Homo sapiens*	Stool sample	BdC	2642081	-	2	58.52	2137	4	56	24	82	3.8	8	0	2	2	TypeIC/TypeIIC	CABKPB0.1
NCTC11816	*Homo sapiens*	Dental caries	BdC	2635828	-	1	58.54	2140	4	56	24	81	3.8	8.2	0	2	2	TypeIC/TypeIIC	LR134349.1

n.p.: not predictable, GH: glycosyl hydrolases, GT: glycosyltransferases, HGT: horizontal gene transfer, RM: restriction–modification.

## Supplementary Materials

Supplementary materials can be found at https://www.mdpi.com/2076-2607/8/11/1720/s1. Table S1. Average nucleotide identity between *B. dentium* strains; Table S2. Prophages of the *B. dentium* taxon; Table S3. CRISPR–Cas systems shared between *B. dentium* strains; Table S4. RM systems among the *B. dentium* taxon; Table S5. Toxin–antitoxin systems of the B. dentium taxon. Figure S1. Carbohydrate utilization by *B. dentium* strains.

## References

[1] Kostic A.D., Howitt M.R., Garrett W.S. (2013). Exploring host-microbiota interactions in animal models and humans. Genes Dev..

[2] Turnbaugh P.J., Ley R.E., Hamady M., Fraser-Liggett C.M., Knight R., Gordon J.I. (2007). The human microbiome project. Nature.

[3] Donovan S.M. (2017). Introduction to the special focus issue on the impact of diet on gut microbiota composition and function and future opportunities for nutritional modulation of the gut microbiome to improve human health. Gut Microbes.

[4] Milani C., Duranti S., Bottacini F., Casey E., Turroni F., Mahony J., Belzer C., Delgado Palacio S., Arboleya Montes S., Mancabelli L. (2017). The First Microbial Colonizers of the Human Gut: Composition, Activities, and Health Implications of the Infant Gut Microbiota. Microbiol. Mol. Biol. Rev. Mmbr..

[5] Lugli G.A., Mangifesta M., Duranti S., Anzalone R., Milani C., Mancabelli L., Alessandri G., Turroni F., Ossiprandi M.C., van Sinderen D. (2018). Phylogenetic classification of six novel species belonging to the genus *Bifidobacterium comprising Bifidobacterium anseris* sp. nov., *Bifidobacterium criceti* sp. nov., *Bifidobacterium imperatoris* sp. nov., *Bifidobacterium italicum* sp. nov., *Bifidobacterium margollesii* sp. nov. and *Bifidobacterium parmae* sp. nov. Syst. Appl. Microbiol..

[6] Lugli G.A., Milani C., Duranti S., Mancabelli L., Mangifesta M., Turroni F., Viappiani A., van Sinderen D., Ventura M. (2018). Tracking the Taxonomy of the Genus Bifidobacterium Based on a Phylogenomic Approach. Appl. Environ. Microbiol..

[7] Duranti S., Mangifesta M., Lugli G.A., Turroni F., Anzalone R., Milani C., Mancabelli L., Ossiprandi M.C., Ventura M. (2017). Bifidobacterium vansinderenii sp. nov., isolated from faeces of emperor tamarin (*Saguinus imperator*). Int. J. Syst. Evol. Microbiol..

[8] Modesto M., Michelini S., Oki K., Biavati B., Watanabe K., Mattarelli P. (2018). Bifidobacterium catulorum sp. nov., a novel taxon from the faeces of the baby common marmoset (*Callithrix jacchus*). Int. J. Syst. Evol. Microbiol..

[9] Modesto M., Puglisi E., Bonetti A., Michelini S., Spiezio C., Sandri C., Sgorbati B., Morelli L., Mattarelli P. (2018). *Bifidobacterium primatium* sp. nov., *Bifidobacterium scaligerum* sp. nov., *Bifidobacterium felsineum* sp. nov. and *Bifidobacterium simiarum* sp. nov.: Four novel taxa isolated from the faeces of the cotton top tamarin (*Saguinus oedipus*) and the emperor tamarin (*Saguinus imperator*). Syst. Appl. Microbiol..

[10] Modesto M., Michelini S., Sansosti M.C., De Filippo C., Cavalieri D., Qvirist L., Andlid T., Spiezio C., Sandri C., Pascarelli S. (2018). Bifidobacterium callitrichidarum sp. nov. from the faeces of the emperor tamarin (*Saguinus imperator*). Int. J. Syst. Evol. Microbiol..

[11] Michelini S., Modesto M., Filippini G., Spiezio C., Sandri C., Biavati B., Pisi A., Mattarelli P. (2018). Corrigendum to “*Bifidobacterium aerophilum* sp. nov., *Bifidobacterium avesanii* sp. nov. and *Bifidobacterium ramosum* sp. nov.: Three novel taxa from the faeces of cotton-top tamarin (*Saguinus oedipus* L.)” [Syst. Appl. Microbiol. 39 (2016) 229–236]. Syst. Appl. Microbiol..

[12] Pechar R., Killer J., Salmonova H., Geigerova M., Svejstil R., Svec P., Sedlacek I., Rada V., Benada O. (2017). *Bifidobacterium apri* sp. nov., a thermophilic actinobacterium isolated from the digestive tract of wild pigs (*Sus scrofa*). Int. J. Syst. Evol. Microbiol..

[13] Alberoni D., Gaggia F., Baffoni L., Modesto M.M., Biavati B., Di Gioia D. (2019). Bifidobacterium xylocopae sp. nov. and Bifidobacterium aemilianum sp. nov., from the carpenter bee (*Xylocopa violacea*) digestive tract. Syst. Appl. Microbiol..

[14] Modesto M., Watanabe K., Arita M., Satti M., Oki K., Sciavilla P., Patavino C., Camma C., Michelini S., Sgorbati B. (2019). *Bifidobacterium jacchi* sp. nov., isolated from the faeces of a baby common marmoset (*Callithrix jacchus*). Int. J. Syst. Evol. Microbiol..

[15] Modesto M., Satti M., Watanabe K., Puglisi E., Morelli L., Huang C.H., Liou J.S., Miyashita M., Tamura T., Saito S. (2019). Characterization of Bifidobacterium species in feaces of the Egyptian fruit bat: Description of *B. vespertilionis* sp. nov. and *B. rousetti* sp. nov. Syst. Appl. Microbiol..

[16] Eckel V.P.L., Ziegler L.M., Vogel R.F., Ehrmann M. (2020). *Bifidobacterium tibiigranuli* sp. nov. isolated from homemade water kefir. Int. J. Syst. Evol. Microbiol..

[17] Duranti S., Lugli G.A., Viappiani A., Mancabelli L., Alessandri G., Anzalone R., Longhi G., Milani C., Ossiprandi M.C., Turroni F. (2020). Characterization of the phylogenetic diversity of two novel species belonging to the genus Bifidobacterium: *Bifidobacterium cebidarum* sp. nov. and *Bifidobacterium leontopitheci* sp. nov. Int. J. Syst. Evol. Microbiol..

[18] Neuzil-Bunesova V., Lugli G.A., Modrackova N., Makovska M., Mrazek J., Mekadim C., Musilova S., Svobodova I., Spanek R., Ventura M. (2020). *Bifidobacterium canis* sp. nov., a novel member of the *Bifidobacterium pseudolongum* phylogenetic group isolated from faeces of a dog (Canis lupus f. familiaris). Int. J. Syst. Evol. Microbiol..

[19] Modesto M., Satti M., Watanabe K., Scarafile D., Huang C.H., Liou J.S., Tamura T., Saito S., Watanabe M., Mori K. (2020). Phylogenetic characterization of two novel species of the genus Bifidobacterium: *Bifidobacterium saimiriisciurei* sp. nov. and *Bifidobacterium platyrrhinorum* sp. nov. Syst. Appl. Microbiol..

[20] Modesto M., Biavati B., Mattarelli P. (2006). Occurrence of the family bifidobacteriaceae in human dental caries and plaque. Caries Res..

[21] Mantzourani M., Fenlon M., Beighton D. (2009). Association between Bifidobacteriaceae and the clinical severity of root caries lesions. Oral Microbiol. Immunol..

[22] Mantzourani M., Gilbert S.C., Fenlon M., Beighton D. (2010). Non-oral bifidobacteria and the aciduric microbiota of the denture plaque biofilm. Mol. Oral Microbiol..

[23] Ventura M., Turroni F., Zomer A., Foroni E., Giubellini V., Bottacini F., Canchaya C., Claesson M.J., He F., Mantzourani M. (2009). The Bifidobacterium dentium Bd1 genome sequence reflects its genetic adaptation to the human oral cavity. PLoS Genet..

[24] Henne K., Rheinberg A., Melzer-Krick B., Conrads G. (2015). Aciduric microbial taxa including Scardovia wiggsiae and Bifidobacterium spp. in caries and caries free subjects. Anaerobe.

[25] Neves B.G., Stipp R.N., Bezerra D.D.S., Guedes S.F.F., Rodrigues L.K.A. (2018). Quantitative analysis of biofilm bacteria according to different stages of early childhood caries. Arch. Oral Biol..

[26] Milani C., Mangifesta M., Mancabelli L., Lugli G.A., James K., Duranti S., Turroni F., Ferrario C., Ossiprandi M.C., van Sinderen D. (2017). Unveiling bifidobacterial biogeography across the mammalian branch of the tree of life. Isme J..

[27] Duranti S., Milani C., Lugli G.A., Turroni F., Mancabelli L., Sanchez B., Ferrario C., Viappiani A., Mangifesta M., Mancino W. (2015). Insights from genomes of representatives of the human gut commensal Bifidobacterium bifidum. Environ. Microbiol..

[28] Duranti S., Milani C., Lugli G.A., Mancabelli L., Turroni F., Ferrario C., Mangifesta M., Viappiani A., Sanchez B., Margolles A. (2016). Evaluation of genetic diversity among strains of the human gut commensal *Bifidobacterium adolescentis*. Sci. Rep..

[29] O’Callaghan A., Bottacini F., O’Connell Motherway M., van Sinderen D. (2015). Pangenome analysis of *Bifidobacterium longum* and site-directed mutagenesis through by-pass of restriction-modification systems. Bmc Genom..

[30] Lugli G.A., Duranti S., Albert K., Mancabelli L., Napoli S., Viappiani A., Anzalone R., Longhi G., Milani C., Turroni F. (2019). Unveiling Genomic Diversity among Members of the Species Bifidobacterium pseudolongum, a Widely Distributed Gut Commensal of the Animal Kingdom. Appl. Environ. Microbiol..

[31] Ventura M., Zink R., Fitzgerald G.F., van Sinderen D. (2005). Gene structure and transcriptional organization of the dnaK operon of *Bifidobacterium breve* UCC 2003 and application of the operon in bifidobacterial tracing. Appl. Environ. Microbiol..

[32] Strandwitz P., Kim K.H., Terekhova D., Liu J.K., Sharma A., Levering J., McDonald D., Dietrich D., Ramadhar T.R., Lekbua A. (2019). GABA-modulating bacteria of the human gut microbiota. Nat. Microbiol..

[33] Duranti S., Ruiz L., Lugli G.A., Tames H., Milani C., Mancabelli L., Mancino W., Longhi G., Carnevali L., Sgoifo A. (2020). *Bifidobacterium adolescentis* as a key member of the human gut microbiota in the production of GABA. Sci. Rep..

[34] Pokusaeva K., Johnson C., Luk B., Uribe G., Fu Y., Oezguen N., Matsunami R.K., Lugo M., Major A., Mori-Akiyama Y. (2017). GABA-producing *Bifidobacterium dentium* modulates visceral sensitivity in the intestine. Neurogastroenterol. Motil. Off. J. Eur. Gastrointest. Motil. Soc..

[35] Engevik M.A., Luk B., Chang-Graham A.L., Hall A., Herrmann B., Ruan W., Endres B.T., Shi Z., Garey K.W., Hyser J.M. (2019). *Bifidobacterium dentium* Fortifies the Intestinal Mucus Layer via Autophagy and Calcium Signaling Pathways. mBio.

[36] Turroni F., Marchesi J.R., Foroni E., Gueimonde M., Shanahan F., Margolles A., van Sinderen D., Ventura M. (2009). Microbiomic analysis of the bifidobacterial population in the human distal gut. Isme J..

[37] Milani C., Lugli G.A., Turroni F., Mancabelli L., Duranti S., Viappiani A., Mangifesta M., Segata N., van Sinderen D., Ventura M. (2014). Evaluation of bifidobacterial community composition in the human gut by means of a targeted amplicon sequencing (ITS) protocol. Fems Microbiol. Ecol..

[38] Lugli G.A., Milani C., Mancabelli L., van Sinderen D., Ventura M. (2016). MEGAnnotator: A user-friendly pipeline for microbial genomes assembly and annotation. Fems Microbiol. Lett..

[39] Bankevich A., Nurk S., Antipov D., Gurevich A.A., Dvorkin M., Kulikov A.S., Lesin V.M., Nikolenko S.I., Pham S., Prjibelski A.D. (2012). SPAdes: A new genome assembly algorithm and its applications to single-cell sequencing. J. Comput. Biol. A J. Comput. Mol. Cell Biol..

[40] Hyatt D., Chen G.L., Locascio P.F., Land M.L., Larimer F.W., Hauser L.J. (2010). Prodigal: Prokaryotic gene recognition and translation initiation site identification. BMC Bioinform..

[41] Zhao Y., Tang H., Ye Y. (2012). RAPSearch2: A fast and memory-efficient protein similarity search tool for next-generation sequencing data. Bioinformatics.

[42] Lowe T.M., Eddy S.R. (1997). tRNAscan-SE: A program for improved detection of transfer RNA genes in genomic sequence. Nucleic Acids Res..

[43] Lagesen K., Hallin P., Rodland E.A., Staerfeldt H.H., Rognes T., Ussery D.W. (2007). RNAmmer: Consistent and rapid annotation of ribosomal RNA genes. Nucleic Acids Res..

[44] Brooks L., Kaze M., Sistrom M. (2019). A Curated, Comprehensive Database of Plasmid Sequences. Microbiol. Resour. Announc..

[45] Arredondo-Alonso S., Rogers M.R.C., Braat J.C., Verschuuren T.D., Top J., Corander J., Willems R.J.L., Schurch A.C. (2018). mlplasmids: A user-friendly tool to predict plasmid- and chromosome-derived sequences for single species. Microb. Genom..

[46] Zhao Y., Wu J., Yang J., Sun S., Xiao J., Yu J. (2012). PGAP: Pan-genomes analysis pipeline. Bioinformatics.

[47] Altschul S.F., Gish W., Miller W., Myers E.W., Lipman D.J. (1990). Basic local alignment search tool. J. Mol. Biol..

[48] Vlietstra W.J., Zielman R., van Dongen R.M., Schultes E.A., Wiesman F., Vos R., van Mulligen E.M., Kors J.A. (2017). Automated extraction of potential migraine biomarkers using a semantic graph. J. Biomed. Inf..

[49] Katoh K., Misawa K., Kuma K., Miyata T. (2002). MAFFT: A novel method for rapid multiple sequence alignment based on fast Fourier transform. Nucleic Acids Res..

[50] Chenna R., Sugawara H., Koike T., Lopez R., Gibson T.J., Higgins D.G., Thompson J.D. (2003). Multiple sequence alignment with the Clustal series of programs. Nucleic Acids Res..

[51] Jain C., Rodriguez R.L., Phillippy A.M., Konstantinidis K.T., Aluru S. (2018). High throughput ANI analysis of 90K prokaryotic genomes reveals clear species boundaries. Nat. Commun..

[52] Lombard V., Golaconda Ramulu H., Drula E., Coutinho P.M., Henrissat B. (2014). The carbohydrate-active enzymes database (CAZy) in 2013. Nucleic Acids Res..

[53] Wheeler T.J., Eddy S.R. (2013). nhmmer: DNA homology search with profile HMMs. Bioinformatics.

[54] Zhang H., Yohe T., Huang L., Entwistle S., Wu P., Yang Z., Busk P.K., Xu Y., Yin Y. (2018). dbCAN2: A meta server for automated carbohydrate-active enzyme annotation. Nucleic Acids Res..

[55] Roberts R.J., Vincze T., Posfai J., Macelis D. (2015). REBASE—A database for DNA restriction and modification: Enzymes, genes and genomes. Nucleic Acids Res.

[56] Couvin D., Bernheim A., Toffano-Nioche C., Touchon M., Michalik J., Neron B., Rocha E.P.C., Vergnaud G., Gautheret D., Pourcel C. (2018). CRISPRCasFinder, an update of CRISRFinder, includes a portable version, enhanced performance and integrates search for Cas proteins. Nucleic Acids Res..

[57] Waack S., Keller O., Asper R., Brodag T., Damm C., Fricke W.F., Surovcik K., Meinicke P., Merkl R. (2006). Score-based prediction of genomic islands in prokaryotic genomes using hidden Markov models. BMC Bioinform..

[58] Milani C., Lugli G.A., Duranti S., Turroni F., Bottacini F., Mangifesta M., Sanchez B., Viappiani A., Mancabelli L., Taminiau B. (2014). Genomic encyclopedia of type strains of the genus Bifidobacterium. Appl. Environ. Microbiol..

[59] Lugli G.A., Milani C., Turroni F., Tremblay D., Ferrario C., Mancabelli L., Duranti S., Ward D.V., Ossiprandi M.C., Moineau S. (2016). Prophages of the genus Bifidobacterium as modulating agents of the infant gut microbiota. Environ. Microbiol..

[60] Arndt D., Grant J.R., Marcu A., Sajed T., Pon A., Liang Y., Wishart D.S. (2016). PHASTER: A better, faster version of the PHAST phage search tool. Nucleic Acids Res..

[61] Lakin S.M., Dean C., Noyes N.R., Dettenwanger A., Ross A.S., Doster E., Rovira P., Abdo Z., Jones K.L., Ruiz J. (2017). MEGARes: An antimicrobial resistance database for high throughput sequencing. Nucleic Acids Res..

[62] Van Heel A.J., de Jong A., Montalban-Lopez M., Kok J., Kuipers O.P. (2013). BAGEL3: Automated identification of genes encoding bacteriocins and (non-)bactericidal posttranslationally modified peptides. Nucleic Acids Res..

[63] Xie Y., Wei Y., Shen Y., Li X., Zhou H., Tai C., Deng Z., Ou H.Y. (2018). TADB 2.0: An updated database of bacterial type II toxin-antitoxin loci. Nucleic Acids Res..

[64] Saeed A.I., Sharov V., White J., Li J., Liang W., Bhagabati N., Braisted J., Klapa M., Currier T., Thiagarajan M. (2003). TM4: A free, open-source system for microarray data management and analysis. BioTechniques.

[65] Tettelin H., Masignani V., Cieslewicz M.J., Donati C., Medini D., Ward N.L., Angiuoli S.V., Crabtree J., Jones A.L., Durkin A.S. (2005). Genome analysis of multiple pathogenic isolates of Streptococcus agalactiae: Implications for the microbial "pan-genome". Proc. Natl. Acad. Sci. USA.

[66] Albert K., Rani A., Sela D.A. (2019). Comparative Pangenomics of the Mammalian Gut Commensal Bifidobacterium longum. Microorganisms.

[67] Bottacini F., O’Connell Motherway M., Kuczynski J., O’Connell K.J., Serafini F., Duranti S., Milani C., Turroni F., Lugli G.A., Zomer A. (2014). Comparative genomics of the Bifidobacterium breve taxon. BMC Genom..

[68] Lugli G.A., Milani C., Turroni F., Duranti S., Mancabelli L., Mangifesta M., Ferrario C., Modesto M., Mattarelli P., Jiri K. (2017). Comparative genomic and phylogenomic analyses of the Bifidobacteriaceae family. BMC Genom..

[69] Lugli G.A., Milani C., Turroni F., Duranti S., Ferrario C., Viappiani A., Mancabelli L., Mangifesta M., Taminiau B., Delcenserie V. (2014). Investigation of the evolutionary development of the genus Bifidobacterium by comparative genomics. Appl. Environ. Microbiol..

[70] Richter M., Rossello-Mora R. (2009). Shifting the genomic gold standard for the prokaryotic species definition. Proc. Natl. Acad. Sci. USA.

[71] Milani C., Lugli G.A., Duranti S., Turroni F., Mancabelli L., Ferrario C., Mangifesta M., Hevia A., Viappiani A., Scholz M. (2015). Bifidobacteria exhibit social behavior through carbohydrate resource sharing in the gut. Sci. Rep..

[72] Botstein D. (1980). A theory of modular evolution for bacteriophages. Ann. N.Y. Acad. Sci..

[73] Bernheim A., Sorek R. (2020). The pan-immune system of bacteria: Antiviral defence as a community resource. Nat. Rev. Microbiol..

[74] Briner A.E., Lugli G.A., Milani C., Duranti S., Turroni F., Gueimonde M., Margolles A., van Sinderen D., Ventura M., Barrangou R. (2015). Occurrence and Diversity of CRISPR-Cas Systems in the Genus Bifidobacterium. PLoS ONE.

[75] Ershova A.S., Rusinov I.S., Spirin S.A., Karyagina A.S., Alexeevski A.V. (2015). Role of Restriction-Modification Systems in Prokaryotic Evolution and Ecology. Biochemistry (Moscow).

[76] Duranti S., Lugli G.A., Mancabelli L., Turroni F., Milani C., Mangifesta M., Ferrario C., Anzalone R., Viappiani A., van Sinderen D. (2017). Prevalence of Antibiotic Resistance Genes among Human Gut-Derived Bifidobacteria. Appl. Environ. Microbiol..

[77] Mancino W., Lugli G.A., Sinderen D.V., Ventura M., Turroni F. (2019). Mobilome and Resistome Reconstruction from Genomes Belonging to Members of the Bifidobacterium Genus. Microorganisms.

