# Supplementary figures and images for: Decoding the Genomic Variability among Members of the Bifidobacterium dentium Species
Source: Microorganisms. 2020 Nov 3;8(11):1720. doi: 10.3390/microorganisms8111720 (PMC7693768; doi:10.3390/microorganisms8111720)

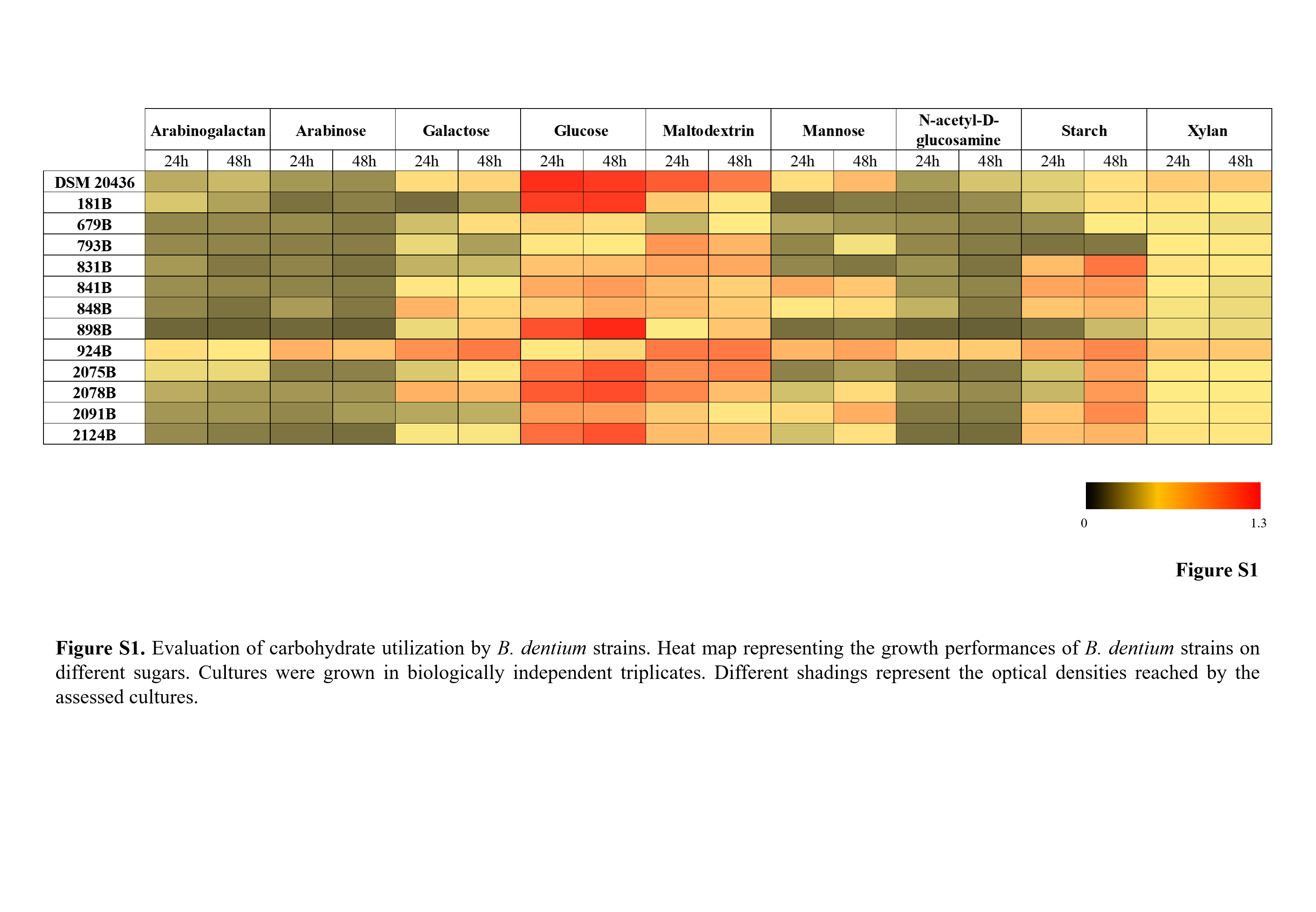

Supplement: Supplementary file 1 [file microorganisms-08-01720-s001.zip › Figure_S1.tif]
